# Spontaneous eye blinking as a diagnostic marker in prolonged disorders of consciousness

**DOI:** 10.1038/s41598-021-01858-3

**Published:** 2021-11-17

**Authors:** Alfonso Magliacano, Martin Rosenfelder, Nina Hieber, Andreas Bender, Anna Estraneo, Luigi Trojano

**Affiliations:** 1 IRCCS Fondazione Don Carlo Gnocchi, Florence, 50143 Italy; 2 Therapiezentrum Burgau, 89331 Burgau, Germany; 3 Ulm University, Institute of Psychology and Pedagogy, Clinical and Biological Psychology, 89081 Ulm, Germany; 4 Department of Neurology, Ludwig-Maximilians-Universität München, 80539 Munich, Germany; 5 SM Della Pietà General Hospital, Neurology Unit, 80035 Nola, Italy; 6 Department of Psychology, University of Campania “Luigi Vanvitelli”, 81100 Caserta, Italy

**Keywords:** Attention, Consciousness, Disorders of consciousness

## Abstract

Clinical diagnosis of patients with prolonged disorders of consciousness is very challenging. As spontaneous eye blink rate (EBR) is reliably correlated with cognitive activity in healthy individuals, we investigated whether EBR could serve as a marker of patients’ level of consciousness. We assessed ten patients in prolonged Vegetative State/Unresponsive Wakefulness Syndrome (VS/UWS; three females; mean age = 50.3 ± 17.8 years) and fourteen patients in Minimally Conscious State (MCS; three females; mean age = 52.9 ± 17.5 years) at their admission to a rehabilitation unit after the acute phase. During two separate 3-min rest conditions, we recorded patients’ EBR by integrating on-line visual and off-line electro-oculographic count. We also assessed EBR during two auditory oddball tasks, i.e. passive listening and active counting of target tones in a sub-group of patients. EBR was significantly higher in MCS than in VS/UWS; moreover, EBR positively correlated with a validated index of responsiveness derived from the Coma Recovery Scale-Revised. Patients’ mean EBR showed no significant differences within sessions and across experimental conditions of the oddball task, in both VS/UWS and MCS. Our findings suggest that, at least in the post-acute phase, observing patients’ EBR for 3 min at rest could help to discriminate between VS/UWS and MCS, improving accuracy of clinical diagnosis.

## Introduction

After a severe brain injury, accurate diagnosis of prolonged (≥ 28 days) disorders of consciousness (DoC)^1^, i.e. Vegetative State/Unresponsive Wakefulness Syndrome (VS/UWS)^2^ and Minimally Conscious State (MCS)^3^, is essential for optimizing patient management^4^. Yet, to date diagnosis of DoC is based on clinical interpretation of patients’ spontaneous behaviour and voluntary responses to multisensory stimulation^5^, that could be however hindered by cognitive and sensory-motor impairments^6^. Indeed, several studies reported that a diagnosis based on clinical consensus might lead to a misdiagnosis rate of 30–40%^6,7^. Therefore, it is of primary importance to identify reliable diagnostic markers of consciousness not relying on patients’ voluntary responses^5^.

Here we focused on spontaneous eye blink rate (EBR) as a possible index of patients’ level of consciousness, easy to detect in clinical practice^8^. EBR is thought to be linked to the activity of the spinal trigeminal complex, whose excitability is modulated by dopaminergic activity in the basal ganglia^9^. According to the well-established mesocircuit hypothesis^10^, dopaminergic projections within striato-thalamo-cortical circuits also play a key role in regulating conscious processes^11–13^ and are a target for therapeutic intervention for recovery of consciousness^12–14^. Indeed, a relative sparing of thalamo-cortical neuronal assemblies has been reported in MCS compared to VS/UWS^15,16^. Moreover, beyond the need for corneal lubrication, EBR is modulated by cognitive variables such as level of vigilance and fatigue^17^, and task demand^17,18^. For instance, during an auditory oddball paradigm, healthy individuals’ spontaneous EBR increases as a function of ‘cognitive load’^19^, i.e. the cognitive resources needed to perform a task.

Driven by the goal to establish additional easy-to-detect markers for improving diagnostic accuracy in patients with DoC, we hypothesized that patients in MCS might present a higher EBR compared to patients in VS/UWS, and that changes in EBR as a function of cognitive load could be observed in patients with a higher level of responsiveness, i.e. in MCS but not in VS/UWS.

To test the above hypotheses, we recorded EBR in a sample of patients with prolonged DoC at rest and, in a sub-group of patients, during an auditory oddball paradigm in which participants were presented with a random sequence of standard and target tones. In order to further manipulate the level of cognitive load, the auditory oddball paradigm was presented with passive and active instructions, and the P300 component on event-related potentials (ERPs) was recorded as an index of patients’ engagement in the tasks^19,20^.

## Methods

### Participants

All patients with DoC due to severe acquired brain injury consecutively admitted to a specialized early rehabilitation unit in Germany from March to December 2020 were screened.

To be eligible for the study patients had to meet the following inclusion/exclusion criteria: (i) clinical diagnosis of VS/UWS or MCS according to standard diagnostic criteria^1,3^, confirmed by repeated (at least two within 2 weeks) assessments by Coma Recovery Scale-Revised (CRS-R^21^), which is the most recommended clinical tool for the diagnosis of DoC, based on the Aspen Workgroup diagnostic criteria^3^ for VS/UWS and MCS^22^; (ii) age 18 to 80 years; (iii) time post-injury (TPI) ≥ 28 days; (iv) lack of previous history of acquired brain injury, psychiatric or neurodegenerative diseases; (v) no craniectomy or scars interfering with an electro-encephalographic (EEG) recording; (vi) stabilized clinical conditions (e.g. no status epilepticus or severe respiratory failure); vii) absence of ophthalmic disorders, or central or peripheral impairments in eyelid motility on clinical evaluation.

A sub-group of patients, who presented a score above 0 at the auditory sub-scale in at least two CRS-R assessments, was assessed on an auditory oddball paradigm, to search for changes in EBR as a function of cognitive load.

### EEG recording and pre-processing

In all patients, an EEG was recorded at rest and during the experimental oddball paradigm (see below) using an EGI NA400 amplifier (Electrical Geodesics Inc./Philips Neuro, Eugene, Oregon, USA) connected to a HydroGel GSN sensor net with 256 channels arranged in an extended 10/20 montage and referenced to the vertex. Impedance was kept below 50 kΩ for electrodes covering the cortex as well as for electro-oculographic (EOG) electrodes, which represented a percentage of 75% of all 256 electrodes. Data were sampled at a 1000-Hz rate and band-pass filtered between 0.1 Hz and 30 Hz; a notch filter was used to eliminate frequencies around 50 Hz for online visualization. If the EEG recording in a session presented a considerable number of artefacts, the acquisition was repeated within the next available day.

Patients’ spontaneous eye blinks were detected by means of a two-fold procedure. During the EEG recording sessions, an experimenter (AM, MR, or NH) inserted a marker within the EEG recording each time she/he clinically observed the patient performing an eye blink; this online procedure allowed to detect eye blinks that would be not identifiable on the recording because of artefacts. After the experimental sessions, the supra- (i.e. channels 37 and 18) and sub-orbital (i.e. channels 241 and 238) channels were extrapolated from the EEG for obtaining a vertical EOG. EOG recordings were visually inspected by a different researcher, blinded with respect to patients’ clinical diagnosis, to check the accuracy of the online detection of eye blinks by comparing the position of the markers with eye blinks on EOG, defined as a sharp positive peak followed by a shallow negative deflection in a time window of < 400 ms^23^. Finally, the EOG recordings were reviewed in random order to reach a final agreement on the number of blinks. EBR was defined as the ratio between the total number of blinks in an experimental condition (rest or oddball) and the duration of the condition in minutes.

For ERPs analysis during the oddball paradigm (see below), the midline EEG channels Fz, Cz and Pz were considered^19,24^, and data were processed using EGI Waveform Tools Package (v5.4.1.2, EGI, Electrical Geodesics Inc., Eugene, Oregon, USA). Responses to target stimuli were segmented over 1000-ms epochs including 100 ms pre-stimulus baseline, time-locked to the stimulus onset. Epochs with voltages exceeding ± 150 μV, eye-movements activity exceeding ± 80 μV and eye-blinks exceeding ± 150 μV were rejected^25^. Accepted epochs were averaged, offline filtered between 0.5 Hz and 30 Hz and corrected for baseline. The P300 wave in response to active counting of target stimuli was defined as a visible positive peak (peak amplitude > 0.75 μV) between 250 and 1000 ms after stimulus onset, and qualitatively identified by means of the visual inspection on the Cz channel^20^. For statistical analysis, peak latency from the time of the stimulus onset and amplitude of the P300 were measured for midline channels (Fz, Cz, Pz) and then averaged across channels.

### Stimuli and task of the oddball paradigm

The auditory oddball paradigm consisted of randomly intermixed tones (non-target: 500 Hz, overall probability: 80%; target: 1000 Hz, overall probability: 20%) delivered through two loudspeakers positioned in front and directed toward the patients (70 dB SPL, 100 ms plateau time, 5 ms rise/fall slope^24^). The auditory tones (78 target and 312 non-target tones) were presented with a 1-s inter-stimulus interval, resulting in a task duration of 8 min. To manipulate the level of cognitive load while controlling for perceptual load^18^, the patients were required to perform the task in two separate sessions: in the first they were required to listen to sounds passively; in the second to actively count target tones. This paradigm has been previously validated in a sample of healthy participants^19^. Stimuli were presented electronically using E-Prime 3.0 software (Psychology Software Tools, Pittsburgh, PA).

### Procedure

In a within-subject design, each patient attended two recording sessions separated by at least 24 h. The sessions were carried out, after customary nursing procedures, between 10 a.m. and 5 p.m., a time period poorly influenced by circadian somnolence peaks in patients with DoC^26^, and during which EBR is thought to be stable in healthy individuals^23^. Both sessions were held at the patient’s bedside, with the patient being supine and with her/his eyes open (i.e. patient being awake), while maintaining a quiet and dimly lit environment. If the patient’s arousal level was insufficient, the arousal facilitation protocol of the CRS-R^21^ was administered; if a patient continued to have her/his eyes closed, the experimental session was postponed to the next available day. Throughout each experimental session, the experimenter was positioned at the patient’s side, in order to be able to observe occurrence of the eye blinks, however beyond the patient’s visual field.

In all patients, EBR was collected during a rest condition (duration: 3 min) in each recording session.

In the sub-group of patients selected for the oddball paradigm (except some of them who could not undergo the complete paradigm due to logistical constraints, largely related to the CoViD-19 pandemic), after the first rest condition a short break was allowed during which, if needed, the patient was briefly stimulated in order to ensure sufficient arousal level. Thereafter, in the first session, the patient performed a passive oddball task (i.e. just listened to the sounds), immediately followed by another rest phase (duration: 3 min). In the second session, after the 3-min rest condition, the patient was required to perform an active oddball task (i.e. to count all the target sounds) and a subsequent 3-min rest phase. The active oddball paradigm was always run in the second session to avoid patients to engage in counting even when not required^27^.

To prevent that awareness of blink recording affected patient’s EBR, throughout both sessions the patient was never informed of the EBR recording, but she/he was simply encouraged to stay relaxed with her/his eyes open, and not to move. This procedure was conducted regardless of patient’s clinical diagnosis.

In order to confirm the patient’s clinical diagnosis and to evaluate the level of responsiveness/consciousness upon the day of each experimental session, a CRS-R was administered by an expert neuropsychologist at the end of each session. The CRS-R with the best total score recorded after the two sessions was considered for the statistical analyses.

For the patients who underwent the complete oddball paradigm, each session lasted about 15 min plus electrode montage, whereas the duration of the CRS-R assessment was about 30 min, resulting in a total duration of 50 min per session approximately (about 35 min for patients who only underwent the rest condition).

### Statistical analyses

The patients’ characteristics (age, sex, etiology, TPI, best CRS-R sub-scores, time between the first and the second session) of the two groups (VS/UWS, MCS) were compared by means of non-parametric Mann–Whitney U or by Chi-square test, as appropriate.

As regards the CRS-R, we recorded the patient’s best behavioural response to single CRS-R items and the total score. However, since some CRS-R total scores (in the range 7–9) might overlap in VS/UWS and MCS^28^, we computed the CRS-R index^29^, a modified score that converts the ordinal CRS-R total score into a linear score considering the highest item in each of the six subscales. The CRS-R index provides information about the patient’s overall level of responsiveness and avoids scores overlapping in the two diagnostic groups.

For analysis of EBR, as a first step, we assessed inter-rater agreement on EBR calculated by the two raters in the two experimental sessions by computing the intraclass correlation coefficients (ICC). Moreover, we measured the inter-measure agreement between EBR detected through online clinical observation and that detected by offline EOG check in the two experimental sessions by computing the ICC. We also calculated patients’ mean variability in EBR between the first and the second session, and compared it between the two groups (VS/UWS, MCS) by means of non-parametric Mann–Whitney U test.

Prior to analysing differences in patients’ EBR at rest across diagnostic groups and experimental sessions, EBR was submitted to Shapiro–Wilk test for normality. As the EBR departed significantly from normality, and also in considerations of the relatively small sample size, we analysed EBR findings by means of non-parametric statistical tests: Mann–Whitney U test to compare two groups; Wilcoxon signed-rank test or Friedman’s test to compare two or three observations, respectively, within groups. Correlations between EBR and continuous variables were evaluated by computing Spearman’s correlation coefficients; correlations between EBR and categorical or ordinal variables were evaluated by computing point-biserial correlation or Kendall’s coefficients, respectively.

As a first step we compared EBR at rest in the two diagnostic groups (VS/UWS, MCS) and in the two sessions (first, second).

For the purpose of correlation analyses, we calculated the average between patients’ raw EBR at rest on the first and on the second session. Then, we computed correlations coefficients between patients’ mean EBR and demographic and clinical variables collected at study entry (sex, age, etiology, TPI, best CRS-R index, CRS-R sub-scores, treatment with dopamine-agonists). Moreover, we evaluated the correlations between patients’ EBR and the CRS-R index collected in each session.

As regards the oddball paradigm, we first investigated possible differences in characteristics (age, sex, etiology, TPI, best CRS-R sub-scores, time between the passive and the active session) of patients who completed the oddball paradigm and the remaining patients of the overall sample.

Then, in the subsample of patients who completed the oddball paradigm, we compared EBR in the two diagnostic groups (VS/UWS), in the two sessions (first, second), and across phases (rest 1, oddball, rest 2). Within this sub-group of patients, we also analyzed the correlation for patients’ EBR collected across the different phases and tasks.

Last, we compared characteristics of the P300 on ERPs (P300 amplitude and latency values) between the two diagnostic groups (VS/UWS, MCS) and between sessions (first, second). Moreover, we checked correlation of patients’ mean EBR with mean P300 amplitude and latency in each task.

The level of significance was set at 0.05 and adjusted for multiple comparisons using the false discovery rate correction. All analyses were performed using IBM SPSS v.25 (IBM Corp., Armonk, New York, USA).

### Ethics

This study was approved by the Ethics Committee of the Medical Faculty at Ludwig-Maximilian University Munich. Written informed consent for the participation in the study was provided by all patients’ surrogate decision-makers. This study complies with Declaration of Helsinki.

## Results

### Patient sample

Out of 63 patients with DoC consecutively screened for the study, we enrolled a convenience sample of 24 patients with prolonged DoC (see Supplementary Fig. S1 online), 10 in VS/UWS and 14 in MCS due to traumatic (n = 7), anoxic (n = 9), or vascular (n = 8) etiologies (Table 1; no case related to CoViD-19).

**Table 1:** Characteristics of the patients.

Patient	Gender	Age (years)	Clinical diagnosis	Etiology	TPI (months)	CRS-R score	CRS-R index	CRS-R assessments (*n*)	Brain lesion location
1*	M	29	VS/UWS	Anoxic	3.9	5 (1–0–2–1-0–1)	4.50	2	NA
2	M	56	VS/UWS	Anoxic	1.6	4 (0–0–2–1-0–1)	3.46	2	Bilateral parieto-occipital
3*	M	62	VS/UWS	Anoxic	1.3	5 (1–0-2–1-0–1)	4.50	4	Global
4	M	38	VS/UWS	TBI	4.8	6 (0–1–2–1-0–2)	4.84	2	Left ICH
5*	F	66	VS/UWS	Anoxic	1.6	6 (1–0–2–1–0–2)	4.84	3	Global, right cerebellum
6	M	50	VS/UWS	Anoxic	1.8	5 (0–0–2–1–0–2)	3.80	3	Global
7	M	65	VS/UWS	Vascular	2.8	8 (2–1-1–2–0–2)	6.92	3	Global
8	F	24	VS/UWS	TBI	10.6	5 (1–0–2–1-0–1)	4.50	5	Right SDH
9	F	77	VS/UWS	Vascular	1.4	6 (1–1–2–1–0–1)	5.54	4	Left parietal, basal ganglia
10	M	36	VS/UWS	Anoxic	1.0	5 (1–0–2–1–0–1)	4.50	5	Global
11	M	23	MCS +	TBI	79.4	9 (3–0–2–1–1–2)	14.22	4	Right parietal
12*	M	68	MCS +	Vascular	1.1	15 (3–4-4–2–0–2)	49.65	2	Right middle cerebral artery
13	M	33	MCS +	TBI	15.2	8 (3–1-1–0-1–2)	13.18	2	Global, right SDH
14*	M	54	MCS-	Anoxic	1.2	10 (2–1-5–1-0–1)	23.26	3	Global, sub-acute infarction right parietal
15*	M	62	MCS-	Anoxic	5.2	10 (2–0-5–1-0–2)	22.55	3	Global
16*	F	57	MCS +	Vascular	1.4	11 (2–3–2–1–1–2)	23.60	2	Right SAH
17*	M	58	MCS-	Vascular	1.6	12 (2–3–5–0–0–2)	39.23	3	Left anterior and middle cerebral artery and basal ganglia
18*	M	60	MCS-	TBI	1.6	14 (2–3–5–2–0–2)	49.65	3	Left SAH, right epidural hematoma; bi-hemispherical contusion bleeding
19*	M	66	MCS-	Anoxic	1.5	10 (2–3–2–1–0–2)	49.65	3	Global
20*	F	48	MCS-	Vascular	2.0	8 (1–3–2–0-0–2)	23.60	3	Left anterior and middle cerebral artery, right anterior cerebral artery, left occipital
21	F	79	MCS +	TBI	4.6	18 (3–5-5–2-1–2)	66.32	5	Right fronto-tempo-parietal
22	M	27	MCS +	Vascular	1.4	18 (4–4–4–3–1–2)	66.32	4	Posterior
23	M	33	MCS +	TBI	3.3	13 (3–5–2–1-0–2)	48.61	4	Bi-hemispherical frontal and basal ganglia, left thalamic, fronto-parietal SDH
24	M	72	MCS-	Vascular	3.0	13 (2–3–5–1-0–2)	40.27	6	right SAH, right SDH

Patients’ best CRS-R total score (and relative sub-scores: auditory-visual-motor-oromotor/verbal-communication-arousal) out of the repeated examination is reported. The asterisk * indicates the patients who completed the full oddball paradigm.

Abbreviations: TPI = Time Post-Injury; CRS-R = Coma Recovery Scale-Revised; M = Male; F = Female; VS/UWS = Vegetative State/Unresponsive Wakefulness Syndrome; MCS = Minimally Conscious State; TBI = Traumatic Brain Injury; NA = Not Available; ICH = Intra-Cerebral Hemorrhage; SDH = Sub-Dural Hemorrhage; SAH = Sub-Arachnoid Hemorrhage.

Two patients in VS/UWS (patients 3 and 6) and one patient in MCS (patient 17), included in the present sample, were on dopamine-agonists (amantadine) when the experimental sessions took place.

For 12/24 patients (50%; 5/10 VS/UWS; 7/14 MCS) at least one experimental session had been postponed due to patients’ agitation or tiredness, or to logistical issues.

The two diagnostic groups did not differ in terms of sex (χ^2^ = 0.23; df = 1; adjusted *p* = 0.78), age (U = 65.0; adjusted *p* = 0.91), etiology (χ^2^ = 3.72; df = 2; adjusted *p* = 0.25), TPI (U = 65.0; adjusted *p* = 0.91), time between sessions (U = 55.0; adjusted *p* = 0.53), and oromotor/verbal (U = 60.5; adjusted *p* = 0.75), communication (U = 45.0; adjusted *p* = 0.24), and arousal (U = 33.0; adjusted *p* = 0.08) CRS-R sub-scores (Table 2). Patients in MCS showed a higher auditory (U = 4.0; adjusted *p* < 0.001), visual (U = 16.0; adjusted *p* = 0.005), and motor (U = 27.5; adjusted *p* = 0.04) CRS-R sub-scores than patients in VS/UWS.

**Table 2:** Patients’ characteristics as a function of clinical diagnosis.

	Total (*n* = 24)	VS/UWS (*n* = 10)	MCS (*n* = 14)	Adjusted *p*
Sex (M/F)	18/6	7/3	11/3	.78
Age (years)	51.8 ± 17.3	50.3 ± 17.8	52.9 ± 17.5	.91
Etiology (TBI/vascular/anoxic)	7/8/9	2/2/6	5/6/3	.25
TPI (months)	6.4 ± 15.9	3.1 ± 2.9	8.7 ± 20.7	.91
CRS-R auditory sub-score	2 (2)	1 (1)	2 (1)	** < .001**
CRS-R visual sub-score	1 (4)	0 (1)	3 (3)	**.005**
CRS-R motor sub-score	2 (3)	2 (0)	4.5 (3)	**.04**
CRS-R oromotor/verbal sub-score	1 (1)	1 (0)	1 (1)	.75
CRS-R communication sub-score	0 (0)	0 (0)	0 (1)	.24
CRS-R arousal sub-score	2 (1)	1 (1)	2 (0)	.08
Time between sessions (days)	5.0 ± 3.9	6.3 ± 5.1	4.1 ± 2.6	.53

Descriptive data are reported as mean ± standard deviation for continuous variables, as median (inter-quartile range) for ordinal variables and as counts for categorical variables. Patients’ best CRS-R index and sub-scores out of the repeated examination is reported. Univariate statistics are based upon the Mann–Whitney U test or χ^2^ test, as appropriate. *p* values was adjusted with the false discovery rate correction. Significant differences are reported in bold.

Abbreviations: VS/UWS = Vegetative State/Unresponsive Wakefulness Syndrome; MCS = Minimally Conscious State; M = Male; F = Female; TBI = Traumatic Brain Injury; TPI = Time Post-Injury; CRS-R = Coma Recovery Scale-Revised.

### Eye blink rate at rest

The inter-rater agreement for patients’ EBR was very high for both the first (ICC for average measure = 0.990 [95% CI = 0.976-0.996]; F_(23,23)_ = 97.07; adjusted *p* < 0.001) and the second (ICC = 0.990 [0.978-0.996]; F_(23,23)_ = 103.28; adjusted *p* < 0.001) session. Similarly, the inter-measure agreement for EBR detected by clinical observation and by EOG inspection was high for both the first (ICC = 0.973 [0.938-0.999]; F_(23,23)_ = 37.31; adjusted *p* < 0.001) and the second (ICC = 0.984 [0.963-0.993]; F_(23,23)_ = 62.06; adjusted *p* < 0.001) session.

**Figure 1:**
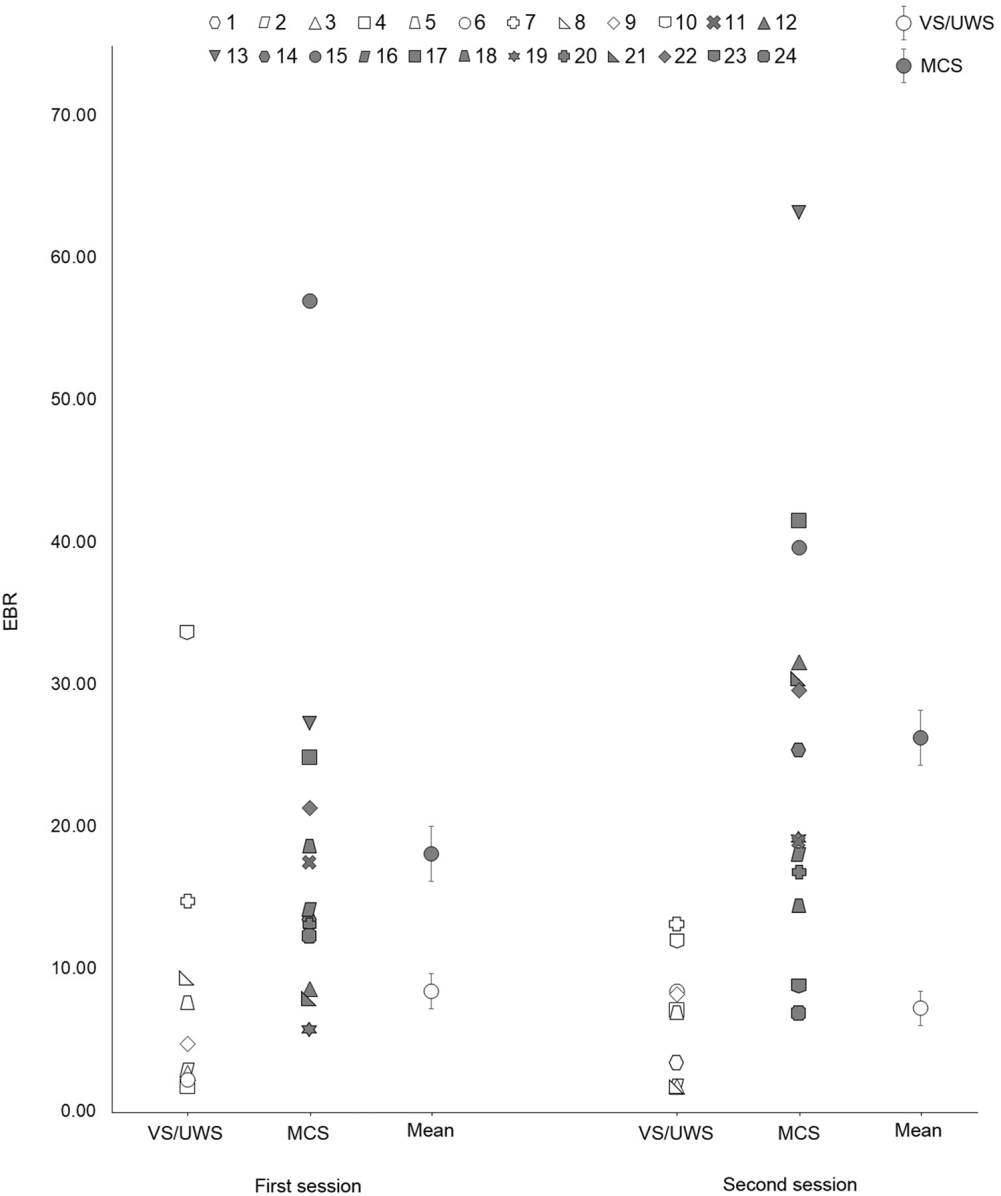
Patients’ mean and individual EBR as a function of the session (first, second) in the two diagnostic groups (VS/UWS, MCS). The void symbols depict patients in VS/UWS, whereas the filled symbols refer to patients in MCS. Error bars display standard errors corrected for within-subject design. Abbreviations: EBR = Eye Blink Rate; VS/UWS = Vegetative State/Unresponsive Wakefulness Syndrome; MCS = Minimally Conscious State.

Raw EBR at rest was consistently higher in MCS than in VS/UWS group; mean variability between the first and second session was 3.8 blink/min (SE = 2.5), with non-significant differences (U = 41.0; adjusted *p* = 0.17) between VS/UWS (mean = -1.7; SE = 2.5) and MCS (mean = 7.8; SE = 3.7; see Fig. 1 for mean and individual EBR in the two sessions).

The analysis on EBR at rest showed a significant difference between diagnostic groups in both the first (VS/UWS: mean = 8.05; SE = 3.10; MCS: mean = 17.99; SE = 3.39; U = 25.0; adjusted *p* = 0.03) and the second session (VS/UWS: mean = 6.35; SE = 1.82; MCS: mean = 25.80; SE = 3.97; U = 8.0; adjusted *p* < 0.001).

EBR did not differ significantly between the two sessions in either the complete sample (Z = -1.44; adjusted *p* = 0.24) or in the single diagnostic sub-groups (VS/UWS: Z = -0.25; adjusted *p* = 0.91; MCS: Z = -1.79; adjusted *p* = 0.16).

**Figure 2:**
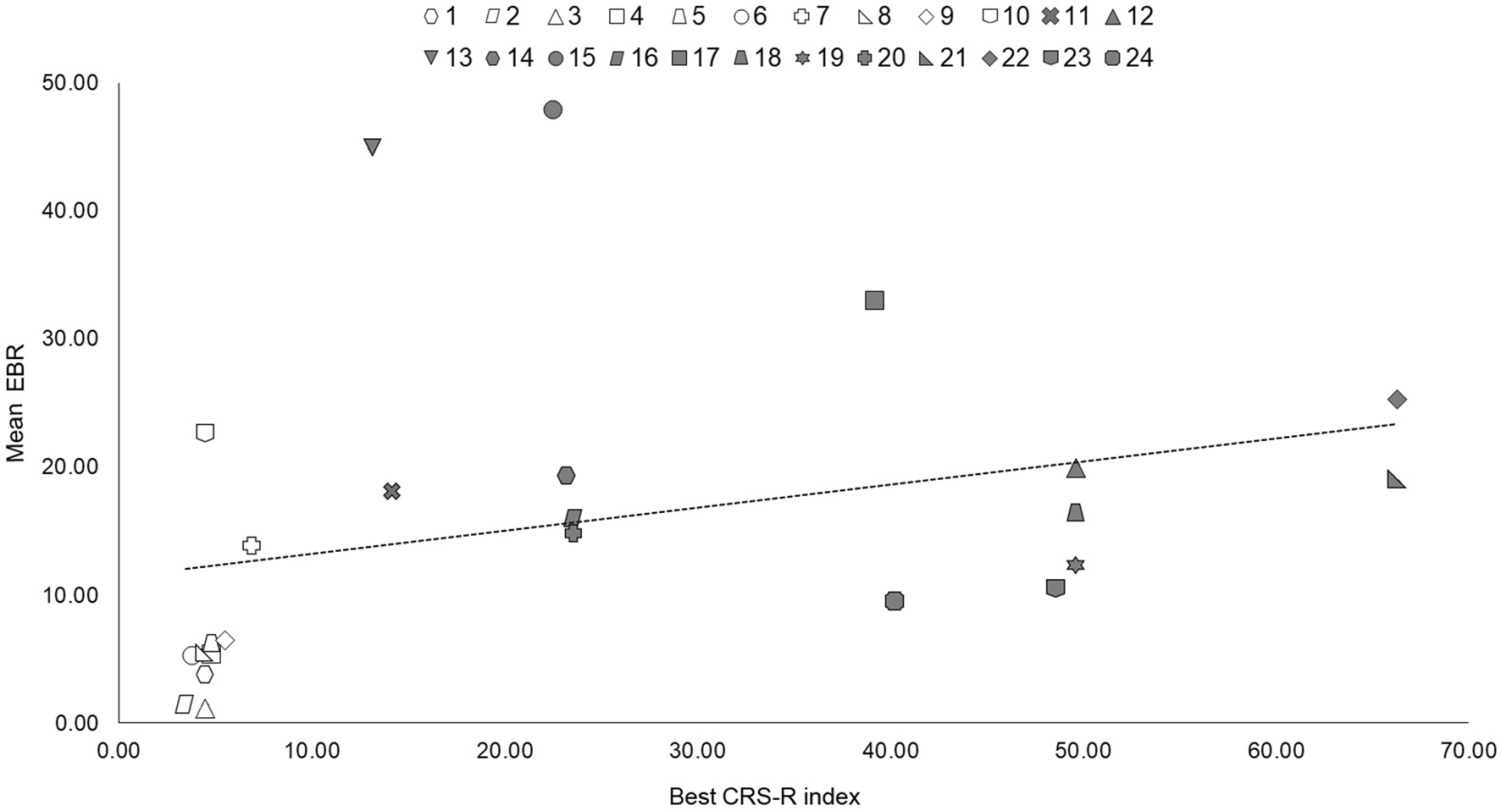
Scatter plot displaying the correlation between patients’ best CRS-R index and mean resting EBR across sessions. The void symbols depict patients in VS/UWS, whereas the filled symbols refer to patients in MCS. Abbreviations: EBR = Eye Blink Rate; CRS-R = Coma Recovery Scale-Revised.

The mean EBR at rest correlated significantly with the best CRS-R index (ρ = 0.60; adjusted *p* = 0.005; Fig. 2), and with the CRS-R auditory sub-score (τ_b_ = 0.58; adjusted *p* < 0.001), whereas no significant correlations were observed with age (ρ = -0.02; adjusted *p* = 0.96), TPI (ρ = -0.04; adjusted *p* = 0.92), sex (r_pb_ = -0.21; adjusted *p* = 0.44), etiology (r_pb_ = -0.13; adjusted *p* = 0.70), dopaminergic therapy (r_pb_ = -0.08; adjusted *p* = 0.86), or with CRS-R visual (τ_b_ = 0.28; adjusted *p* = 0.15), motor (τ_b_ = 0.29; adjusted *p* = 0.17), oromotor/verbal (τ_b_ = 0.08; adjusted *p* = 0.77), communication (τ_b_ = 0.35; adjusted *p* = 0.11), and arousal (τ_b_ = 0.33; adjusted *p* = 0.14) sub-scores.

The correlation between the EBR collected in the first session and the respective CRS-R index did not reach statistical significance (ρ = 0.40; adjusted *p* = 0.08), whereas the EBR collected in the second session significantly correlated with the CRS-R index collected in the second session (ρ = 0.68; adjusted *p* < 0.001).

### Eye blink rate as a function of cognitive load

Out of the overall sample, three patients (patients 2, 4, 6 in VS/UWS) were excluded from the oddball paradigm due to their absence of response to the CRS-R auditory sub-scale. Patients 7–10 in VS/UWS and patients 11 and 21–24 in MCS were also excluded due to constraints related to the current CoViD-19 pandemic. Patient 13 in MCS underwent the full paradigm but was excluded from the analysis due to low arousal during the first session, and the impossibility to perform recording in following days. Therefore, 11 patients (patients 1, 3, 5 in VS/UWS; and patients 12, 14–20 in MCS; see Table 1) completed the whole paradigm including the oddball task.

This sub-group of patients did not differ from the remaining patients of the overall sample in any demographic, anamnestic or clinical characteristic (all adjusted *p* > 0.05).

**Figure 3:**
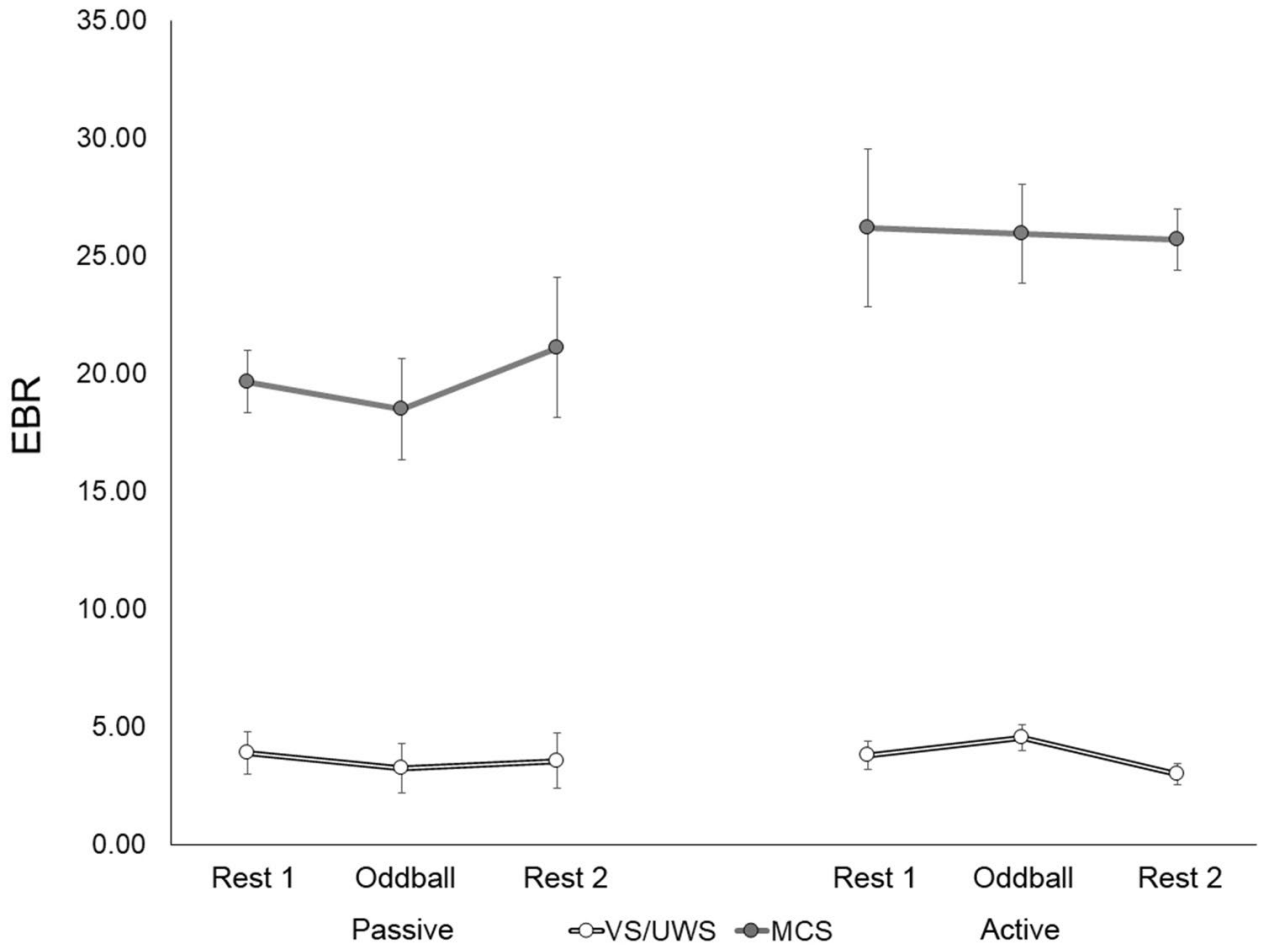
Patients’ (n = 11) EBR as a function of the task (passive, active) and the phase (Rest 1, Oddball, Rest 2) in the two diagnostic groups (VS/UWS, MCS). Error bars display standard errors corrected for within-subject design. EBR was significantly different between diagnostic groups (adjusted *p* = .04), but no significant differences were observed within groups across tasks and phases (all adjusted *p* > .05). Abbreviations: EBR = Eye Blink Rate; VS/UWS = Vegetative State/Unresponsive Wakefulness Syndrome; MCS = Minimally Conscious State.

Considering data from all patients across sessions, mean EBR significantly differed between the two diagnostic groups (U = 0.0; adjusted *p* = 0.04), as it was higher in MCS (mean = 22.00; SE = 4.75) than in VS/UWS (mean = 3.70; SE = 1.46). EBR did not differ significantly between the two sessions either in the whole DoC group (Z = -1.51; adjusted *p* = 0.22) or in single diagnostic sub-groups (VS/UWS: Z = -1.07; adjusted *p* = 0.41; MCS: Z = -1.26; adjusted *p* = 0.31). Similarly, EBR did not differ significantly across the three phases of the task in either the whole group (χ^2^ = 2.36; df = 2; adjusted *p* = 0.43) or in single diagnostic sub-groups (VS/UWS: χ^2^ = 0.67; df = 2; adjusted *p* = 0.86; MCS: χ^2^ = 3.00; df = 2; adjusted *p* = 0.33; Fig. 3).

Concerning data from all patients within sessions, EBR was higher in patients in MCS than in patients in VS/UWS in both sessions, but the difference between the two groups reached the significance threshold in the second session only (first session: U = 2.0; adjusted *p* = 0.12; second session: U = 0.0; adjusted *p* = 0.04). Moreover, patients in MCS showed a significantly higher EBR than patients in VS/UWS in all the three phases of the task (all adjusted *p* < 0.05).

EBR collected in each phase of the two sessions correlated significantly with EBR collected in the other phase within the same session, but not across sessions (see Supplementary Table S1 online).

### Event-related potentials

Analysis of P300 was performed on a sub-sample of 10 patients, since EEG recording from one patient (patient 19) was excluded due to presence of artefacts.

A visually detectable P300 in response to target stimuli was observed only in three MCS patients (patients 14, 16, 18). Mean number of epochs accepted for analysis of P300 was 187 ± 119 (range: 36–367) in the passive task and 169 ± 112 (range: 50–387) in the active task.

The two diagnostic groups did not differ significantly in amplitude (U = 5.0; adjusted *p* = 0.24) or latency (U = 11.0; adjusted *p* = 0.92) of the ERPs. Similarly, there were no significant differences across sessions in both amplitude (Z = 0.00; adjusted *p* = 0.99) or latency (Z = -1.69; adjusted *p* = 0.17; see Supplementary Table S2 online for patients’ mean amplitude and latency values).

No significant correlation was found between P300 amplitude or latency and EBR in either task (all adjusted *p* > 0.05).

## Discussion

In the present cross-sectional study, we observed a significantly higher EBR in the MCS than in the VS/UWS group. In a sub-group of patients, the experimental manipulation of tasks’ cognitive load did not exert any effect on patients’ EBR irrespective of clinical diagnosis. At group level, patients’ mean EBR was found to be quite stable across phases within each experimental session. Nonetheless, some variability was observed across sessions at individual level in both VS/UWS and MCS, and might be ascribed to small fluctuations in arousal and/or fatigue^17^.

The difference in EBR between VS/UWS and MCS could not be ascribed to demographic or anamnestic characteristics (i.e. age, sex, etiology, and TPI), that did not differ between the two diagnostic groups and did not correlate with EBR. Similarly, it is unlikely that differences in EBR between the two groups could be explained by the arousal level, as the CRS-R arousal sub-score did not differ significantly between VS/UWS and MCS patients and did not correlate significantly with EBR. Moreover, differences in EBR between VS/UWS and MCS could not be explained by pharmacological treatment, as in the three patients (two in VS/UWS; one in MCS), who were treated with dopaminergic drugs during the study, EBR did not differ from other patients of their respective diagnostic group.

It is well accepted that spontaneous eye blinks not only serve the lubrication of the corneal surface, but are affected by several variables including cognitive states^9^. Indeed, three or four blinks/min are thought to be sufficient for preserving the corneal tear film^30^, whereas mean EBR of healthy individuals is usually higher^8^. In our study, the considerably low EBR shown by patients in VS/UWS seemed to be very similar to that necessary for maintaining eye hydration, and might be interpreted as a basic spontaneous physiologic reflex triggered by dry spots appearing on the corneal epithelium^31^, with little or no additional influence of cognitive activity.

The neurobiological factor underlying the significantly higher spontaneous EBR in MCS than in VS/UWS is probably represented by dopaminergic activity. Indeed, there is evidence that dopamine modulates EBR in healthy and brain-damaged individuals^32^. Dopamine has a critical role in controlling arousal and attentional systems^33^, too, and several studies linked a higher EBR to higher attentional resources involved in a cognitive activity^17–19^. On this basis, we could speculate that the higher EBR in MCS, compared to VS/UWS, is an index of patients’ relatively spared cortico-subcortical dopaminergic projections^34^, which are likely related to the minimal but definite behavioural evidence of self and environment awareness in MCS^3^.

In the present study, we reduced the EEG montage time and the discomfort for patients by using an EEG cap rather than employing single electrodes. Nonetheless, the experimental setting and the montage of EEG cap could still induce some level of discomfort in patients. The higher EBR observed in MCS with respect to VS/UWS might be interpreted as related to a higher sensitivity of patients in MCS to distress, and thus could inform about the level of responsiveness rather than directly reflect different levels of consciousness. Future studies could test this alternative hypothesis by comparing patients’ EBR collected by EEG and by clinical observation on different sessions.

On a behavioural level, patients in MCS exhibit a greater tendency to visually explore the surrounding environment, in comparison to patients in VS/UWS. Indeed, an intentional behaviour frequently shown by patients in MCS consists in visually pursuing and/or fixating environmental or salient stimuli^3,35^. The visual exploration behaviour of patients in MCS might involve a broad scan of the environment through several large saccades^36^, which are often accompanied by blinks^37^, thus contributing to increase EBR in MCS. Moreover, in healthy individuals spontaneous blinking at rest is thought to improve environmental monitoring by systematically comparing the image appearing at the eyelid opening with its mnestic trace stored at the moment of eyelid closure^38^, in line with the so-called “sentinel theory”^39^. Interestingly, electrophysiological studies identified EEG delta and alpha activity phase- and time-locked to spontaneous blinking (i.e. blink-related oscillations, BRO) in healthy individuals, which is correlated to activity in the posterior cingulate cortex/precuneus (PCC/PCu)^38,40^. Previous studies showed that delta BRO are very low in both VS/UWS and MCS patients^41^, whereas alpha BRO are lower in VS/UWS than in MCS and healthy controls, with patients in MCS showing an intermediate signal intensity on the PCC/PCu with respect to VS/UWS and healthy controls^40^. We could not collect these electrophysiological measures in the present study, but searched for relationships between EBR and the CRS-R visual sub-scale, which includes items for assessing visual pursuit and fixation (associated with visual exploration behaviour). Although patients in VS/UWS and MCS differed significantly in their CRS-R visual sub-scores, the correlation between EBR and the CRS-R visual sub-score did not reach the significance level. This was probably related to the presence of other unexplored factors influencing EBR, i.e. in one patient in MCS (patient 15) who did not exhibit any visual response at the repeated CRS-R in spite of a high EBR. Nonetheless, it is plausible that the visual exploration behavior and BRO might help to explain the higher EBR in MCS compared to VS/UWS, although these considerations should be tested specifically.

It is also important to consider that the CRS-R visual sub-scale includes an item assessing the blink reflex to visual threat. The lack of significant correlation between EBR and the CRS-R visual sub-score could also be explained by the difference in the neural mechanisms controlling blink reflex and spontaneous blinking. Indeed, blink reflex is elicited by multisensory inputs through the brainstem reticular formation to the facial nerve nucleus or adjacent structures^42^, whereas spontaneous blinking is thought to be modulated by dopaminergic activity in the basal ganglia^9^ and related with activity in several cortical regions, particularly those involved in the brain default mode network^43,44^.

The role of EBR as a supplementary source of information in discriminating between diagnostic categories of prolonged DoC was supported by the significant positive correlation found between EBR and the CRS-R index that, as recalled above, provides reliable information about the patient’s overall level of responsiveness^29^. However, this correlation was stronger for data collected during the second than during the first session, likely in relation to small fluctuations in arousal and/or fatigue in patients in MCS, not significant at a group level.

Our findings are partly similar to those reported by Bonfiglio et al.^45^ in a sample of thirteen patients with DoC classified as being in ‘persistent vegetative state’ (PVS; roughly corresponding to our patients in VS/UWS) or not (non-PVS; including patients in MCS and patients who were emerging from MCS) based on their score at the Glasgow Outcome Scale (GOS)^46^. Mean resting EBR at study entry collected in Bonfiglio et al.’s non-PVS patients (around 15 blink/min) did not differ substantially from that we recorded in our patients in MCS (it was a little higher in our patients, likely in relation to the recording setting: our patients wore an EEG cap, Bonfiglio et al.’s patients did not). At variance, mean EBR differed markedly between Bonfiglio et al.’s patients in PVS (who showed the highest values in the study, around 17 blink/min, similar to EBR exhibited by our patients in MCS) and our patients in VS (who showed the lowest values). It is hard to reconcile these different findings, but this apparent discrepancy could be explained by differences in diagnostic criteria (GOS in Bonfiglio et al., with possible misdiagnosis of patients in MCS, vs. CRS-R here) and in time post-injury. Indeed, Bonfiglio et al. examined a quite heterogeneous sample of patients in VS (mean months after onset: 16.7; median: 6.5; range: 1–72; only one patient assessed at 2 months post-onset, and the others at 6 or more) whereas our sample mostly included patients at their first admission in a rehabilitation unit after the acute phase post-injury (mean months after onset: 3.1; median: 1.7; range: 1–10; all patients but one assessed within 5 months post-onset). As in their study^45^ Bonfiglio et al. observed significant changes of EBR during hospitalization (i.e. in 2–3 months after study entry) in some patients, the huge differences in TPI in the two samples of patients in VS/UWS might really play a relevant role in explaining the differences between the studies. Therefore, the potential clinical diagnostic implications of EBR and its evolution over time in DoC, also as a function of the clinical diagnosis, need further evidence to be fully comprehended.

The auditory oddball paradigm used here has been found to increase the EBR as a function of the task’s cognitive load in healthy participants^19^. Here this effect could not be observed: patients’ EBR remained stable in the rest and the oddball conditions, as well as in the passive and the active versions of the oddball task. Albeit this could have been expected for patients in VS/UWS, this finding in MCS was not consistent with our initial hypothesis. A change in EBR depending on the cognitive load would have demonstrated that the patients were performing the task correctly. These findings would therefore suggest that most patients in our sample were not performing or were not able to perform the task, thus making the manipulation of cognitive load ineffective. This inference was supported by ERPs data. Previous evidence showed that ERPs, and specifically the P300, could provide an online indication of cortical processing in DoC^47^, and differences in the P300 amplitude and latency between passive and active tasks could be interpreted as a sign that patients are voluntarily trying to perform the task^20^. Instead, the present results showed no significant differences in the characteristics of the P300 between the passive and the active condition of the oddball task, even in patients showing language-related behaviours (i.e. patients in MCS+^48^: patients 12 and 16). Moreover, we did not find a significant correlation between P300 characteristics and EBR. In this respect it is worth mentioning that both P300 and EBR are supposed to be indices of attention and to be modulated by dopaminergic activity, but P300 and EBR could be controlled by different mechanisms. In particular, EBR seems to depend on a wide central dopaminergic system^32^, whereas P300 could be mediated by a specific mesocortico-limbic dopaminergic action^49^. Accordingly, a recent study on healthy participants showed that EBR did not correlate with the P300 recorded during an oddball or a Go/No-Go^50^ task. Taken together, ERPs data were consistent with the idea that patients of either group did not perform the task as requested.

The accomplishment of the oddball paradigm might be potentially hindered by co-occurrence of cognitive impairments. Indeed, here we use the umbrella term ‘cognitive load’ to define the attentional and cognitive demands required by the oddball tasks compared to rest, and in particular by the active compared to the passive task. However, the active oddball task actually implied several cognitive processes, such as sustained attention (i.e. trying not to miss any tone^51^), selective attention (i.e. distinguishing target from non-target tones^52^), and working memory (i.e. keeping in mind the count of target tones^53^). Each of these processes can be selectively impaired in severely brain-injured patients, and our paradigm could not allow to differentiate among them. Moreover, it should be considered the possible presence of aphasia in some patients, and in particular those with a brain lesion to left hemisphere (e.g. patients 17, 18, 20), which could have led to inability to understand task instructions. Further studies should investigate whether the expected effect of cognitive load could be identified in patients with Locked-In Syndrome (LIS; i.e. full consciousness but severe or complete motor impairment), and whether it would help in discriminating LIS from DoC^54^.

This study has several possible limitations. First, blindness to patients’ clinical diagnosis could not be assured for the researcher who evaluated EBR during the experimental sessions. However, we reduced the possible examiner-related bias by means of an off-line EOG check performed by two independent researchers with high inter-rater agreement.

Second, here we used a fixed order of the tasks, with the passive task always preceding the active task. This procedure was based on previous literature on healthy individuals^19,55,56^ and on patients with DoC^20,27,57^ suggesting that performing the active before the passive condition could trigger attending the stimuli also when this is not requested. In the present study the two conditions were separated by some days, with a possible reduction of such a carry-over effect, but we preferred to employ the same procedure previously validated on healthy individuals^19^.

Third, we investigated EBR but we did not focus on the description of further characteristics of patients’ eye blinks (e.g. blink amplitude, variability in intervals between blinks) due to technical constraints.

Fourth, even if we performed experimental sessions in a time of the day poorly affected by somnolence peaks, some studies suggested that significant differences in patients’ responsiveness can be observed between morning and afternoon^1,58,59^. Performing both recording sessions at the same time of the day in all patients could have limited this possible bias, but was not feasible because of the specific therapeutic needs of the single patients. Circadian changes in EBR in patients with DoC remain to be fully explored.

Fifth, previous studies^60^ suggested that up to the fourth CRS-R assessment, behavioural fluctuations may still impact the diagnostic accuracy. Although here patients’ diagnosis was based on clinical evaluation by expert neuropsychologists supported by repeated (at least two and up to six) CRS-R assessments, some patients underwent less than five CRS-R, resulting in a possible misdiagnosis rate ranging from 9 to 24%^60^.

Finally, we are aware that the results of the present study are based on a relatively small sample size, and are to be interpreted with caution. Moreover, the small sample size did not allow us to perform solid analyses within the two diagnostic groups, for instance comparing patients in MCS as a function of complexity of their behaviour (i.e. MCS- and MCS+)^48^. Future studies should enrol larger samples to unravel these issues and to confirm the present results, ascertaining possible sensitivity and specificity of different EBR values in distinguishing VS/UWS from MCS.

In conclusion, we demonstrated that spontaneous EBR was significantly higher in MCS than in VS/UWS and correlated positively with patients’ overall level of responsiveness as measured by the CRS-R index. These findings represent an evidence that, at least at their admission at a rehabilitation unit after the acute phase, EBR might constitute a diagnostic biomarker for prolonged DoC, easily to be collected at bedside, providing additional information when some CRS-R sub-scales could not detect conscious behaviours due to patient’s sensory or motor deficits.

If our results will hold true in larger study populations, a simple and low-burden but decisive clinical marker, such as counting eye blinks over 3 min, will help to reduce rate of misdiagnosis in DoC patients, without the need for additional high-tech methods.

## Supplementary Information

Supplementary Information.

## Data Availability

The datasets generated during and/or analysed during the current study are available from the corresponding author on reasonable request.

## References

[1] Giacino JT (2018). Practice guideline update recommendations summary: Disorders of consciousness. Neurology.

[2] Laureys S (2010). Unresponsive wakefulness syndrome: A new name for the vegetative state or apallic syndrome. BMC Med..

[3] Giacino JT (2002). The minimally conscious state: Definition and diagnostic criteria. Neurology.

[4] Estraneo, A. & Trojano, L. Prognosis in disorders of consciousness. In *Coma and disorders of consciousness* 17–36 (Springer, 2018).

[5] Kondziella D (2020). European Academy of Neurology guideline on the diagnosis of coma and other disorders of consciousness. Eur. J. Neurol..

[6] Majerus S, Gill-Thwaites H, Andrews K, Laureys S (2005). Behavioral evaluation of consciousness in severe brain damage. Prog. Brain Res..

[7] Schnakers C (2009). Diagnostic accuracy of the vegetative and minimally conscious state: Clinical consensus versus standardized neurobehavioral assessment. BMC Neurol..

[8] McMonnies, C. W. Blinking Mechanisms. *Encycl. Eye* 202–208 (2010).

[9] Kaminer J, Powers AS, Horn KG, Hui C, Evinger C (2011). Characterizing the spontaneous blink generator: An animal model. J. Neurosci..

[10] Schiff ND (2010). Recovery of consciousness after brain injury: A mesocircuit hypothesis. Trends Neurosci..

[11] Adams JH (2000). The neuropathology of the vegetative state after an acute brain insult. Brain.

[12] Thibaut A, Schiff N, Giacino J, Laureys S, Gosseries O (2019). Therapeutic interventions in patients with prolonged disorders of consciousness. Lancet. Neurol..

[13] Masotta, O., Trojano, L., Loreto, V., Moretta, P. & Estraneo, A. Selegiline in patients with disorder of consciousness: An open pilot study. *Can. J. Neurol. Sci. Le J. Can. des Sci. Neurol.* **45**, 688–691 (2018). 10.1017/cjn.2018.315 30430963

[14] Edlow BL (2021). Therapies to restore consciousness in patients with severe brain injuries: A gap analysis and future directions. Neurocrit. Care.

[15] Annen J (2018). Regional brain volumetry and brain function in severely brain-injured patients. Ann. Neurol..

[16] Rubeaux, M. *et al.* Thalamic volume as a biomarker for disorders of consciousness. *10th Int. Symp. Med. Inf. Process. Anal.* **9287**, 92870R (2015).

[17] Huang Z, Stanford MS, Barratt ES (1994). Blink rate related to impulsiveness and task demands during performance of event-related potential tasks. Pers. Individ. Dif..

[18] Chen S, Epps J (2014). Using task-induced pupil diameter and blink rate to infer cognitive load. Hum.-Comput. Interact..

[19] Magliacano A, Fiorenza S, Estraneo A, Trojano L (2020). Eye blink rate increases as a function of cognitive load during an auditory oddball paradigm. Neurosci. Lett..

[20] Risetti M (2013). On ERPs detection in disorders of consciousness rehabilitation. Front. Hum. Neurosci..

[21] Giacino JT, Kalmar K, Whyte J (2004). The JFK Coma Recovery Scale-Revised: Measurement characteristics and diagnostic utility. Arch. Phys. Med. Rehabil..

[22] Seel RT (2010). Assessment scales for disorders of consciousness: Evidence-based recommendations for clinical practice and research. Arch. Phys. Med. Rehabil..

[23] Barbato G (2000). Diurnal variation in spontaneous eye-blink rate. Psychiatry Res..

[24] Duncan CC (2009). Event-related potentials in clinical research: Guidelines for eliciting, recording, and quantifying mismatch negativity, P300, and N400. Clin. Neurophysiol..

[25] Faugeras F (2012). Event related potentials elicited by violations of auditory regularities in patients with impaired consciousness. Neuropsychologia.

[26] Wislowska M (2017). Night and day variations of sleep in patients with disorders of consciousness. Sci. Rep..

[27] Morlet D, Ruby P, André-Obadia N, Fischer C (2017). The auditory oddball paradigm revised to improve bedside detection of consciousness in behaviorally unresponsive patients. Psychophysiology.

[28] Sattin, D. *et al.* The Coma Recovery Scale Modified Score: a new scoring system for the Coma Recovery Scale-revised for assessment of patients with disorders of consciousness. *Int. J. Rehabil. Res. Int. Zeitschrift fur Rehabil. Rev. Int. Rech. Readapt.* **38**, 350–356 (2015). 10.1097/MRR.0000000000000135 26465775

[29] Annen, J. *et al.* Diagnostic accuracy of the CRS-R index in patients with disorders of consciousness. *Brain Inj.* 1–4 (2019). 10.1080/02699052.2019.1644376 31319707

[30] Al-Abdulmunem M (1999). Relation between tear breakup time and spontaneous blink rate. Int. Contact Lens Clin..

[31] Holly FJ (1973). Formation and rupture of the tear film. Exp. Eye Res..

[32] Jongkees BJ, Colzato LS (2016). Spontaneous eye blink rate as predictor of dopamine-related cognitive function - A review. Neurosci. Biobehav. Rev..

[33] Thiele A, Bellgrove MA (2018). Neuromodulation of attention. Neuron.

[34] Jennett B, Adams JH, Murray LS, Graham DI (2001). Neuropathology in vegetative and severely disabled patients after head injury. Neurology.

[35] Trojano L (2012). Quantitative assessment of visual behavior in disorders of consciousness. J. Neurol..

[36] Gameiro RR, Kaspar K, König SU, Nordholt S, König P (2017). Exploration and exploitation in natural viewing behavior. Sci. Rep..

[37] Rottach KG (1998). Properties of horizontal saccades accompanied by blinks. J. Neurophysiol..

[38] Bonfiglio L (2011). Reciprocal dynamics of EEG alpha and delta oscillations during spontaneous blinking at rest: A survey on a default mode-based visuo-spatial awareness. Int. J. Psychophysiol..

[39] Buckner RL, Andrews-Hanna JR, Schacter DL (2008). The brain’s default network: Anatomy, function, and relevance to disease. Ann. N. Y. Acad. Sci..

[40] Bonfiglio L (2014). Spectral parameters modulation and source localization of blink-related alpha and low-beta oscillations differentiate minimally conscious state from vegetative state/unresponsive wakefulness syndrome. PLoS ONE.

[41] Bonfiglio L (2013). Cortical source of blink-related delta oscillations and their correlation with levels of consciousness. Hum. Brain Mapp..

[42] Miwa H, Nohara C, Hotta M, Shimo Y, Amemiya K (1998). Somatosensory-evoked blink response: Investigation of the physiological mechanism. Brain.

[43] Nakano T, Kato M, Morito Y, Itoi S, Kitazawa S (2013). Blink-related momentary activation of the default mode network while viewing videos. Proc. Natl. Acad. Sci. USA.

[44] Nakano T (2017). The right angular gyrus controls spontaneous eyeblink rate: A combined structural MRI and TMS study. Cortex.

[45] Bonfiglio L (2005). Spontaneous blinking behaviour in persistent vegetative and minimally conscious states: Relationships with evolution and outcome. Brain Res. Bull..

[46] Jennett B, Bond M (1975). Assessment of outcome after severe brain damage. Lancet.

[47] Kotchoubey B, Lang S, Bostanov V, Birbaumer N (2002). Is there a mind? Electrophysiology of unconscious patients. News Physiol. Sci..

[48] Bruno MA, Vanhaudenhuyse A, Thibaut A, Moonen G, Laureys S (2011). From unresponsive wakefulness to minimally conscious PLUS and functional locked-in syndromes: Recent advances in our understanding of disorders of consciousness. J. Neurol..

[49] Beste C, Willemssen R, Saft C, Falkenstein M (2010). Response inhibition subprocesses and dopaminergic pathways: Basal ganglia disease effects. Neuropsychologia.

[50] Zhang T (2016). Different relationships between central dopamine system and sub-processes of inhibition: Spontaneous eye blink rate relates with N2 but not P3 in a Go/Nogo task. Brain Cogn..

[51] Ciria LF, Perakakis P, Luque-casado A, Morato C, Sanabria D (2017). The relationship between sustained attention and aerobic fitness in a group of young adults. PeerJ.

[52] Kemp AH (2010). Impact of depression heterogeneity on attention: An auditory oddball event related potential study. J. Affect. Disord..

[53] George EM, Coch D (2011). Music training and working memory: An ERP study. Neuropsychologia.

[54] Trojano L, Moretta P, Estraneo A, Santoro L (2010). Neuropsychologic assessment and cognitive rehabilitation in a patient with locked-in syndrome and left neglect. Arch. Phys. Med. Rehabil..

[55] Mertens R, Polich J (1997). P300 from a single-stimulus paradigm: passive versus active tasks and stimulus modality. Electroencephalogr. Clin. Neurophysiol..

[56] Wronka E, Kaiser J, Coenen AML (2008). The auditory P3 from passive and active three-stimulus oddball paradigm. Acta Neurobiol. Exp..

[57] Schettini, F. *et al.* P300 latency Jitter occurrence in patients with disorders of consciousness: Toward a better design for Brain Computer Interface applications. *Annu. Int. Conf. IEEE Eng. Med. Biol. Soc. IEEE Eng. Med. Biol. Soc. Annu. Int. Conf.* **2015**, 6178–6181 (2015). 10.1109/EMBC.2015.7319803 26737703

[58] Cortese MD (2015). Coma recovery scale-r: variability in the disorder of consciousness. BMC Neurol..

[59] Candelieri A, Cortese MD, Dolce G, Riganello F, Sannita WG (2011). Visual pursuit: within-day variability in the severe disorder of consciousness. J. Neurotrauma.

[60] Wannez S, Heine L, Thonnard M, Gosseries O, Laureys S (2017). The Repetition of Behavioral Assessments in Diagnosis of Disorders of Consciousness. ANN NEUROL.

