# Supplementary material for: Spontaneous eye blinking as a diagnostic marker in prolonged disorders of consciousness
Source: Sci Rep. 2021 Nov 17;11:22393. doi: 10.1038/s41598-021-01858-3 (PMC8599689; doi:10.1038/s41598-021-01858-3)
Supplement: Supplementary file 1 — Supplementary Information. [file 41598_2021_1858_MOESM1_ESM.docx]

**Spontaneous eye blinking as a diagnostic marker in prolonged disorders of consciousness**

Alfonso Magliacano^1,+^, Martin Rosenfelder^2,3,+^, Nina Hieber^2,3^, Andreas Bender^2,4^, Anna Estraneo^1,5^, Luigi Trojano^6,*^

^1^IRCCS Fondazione Don Carlo Gnocchi, Florence, 50143, Italy

^2^Therapiezentrum Burgau, Burgau, 89331, Germany

^3^Ulm University, Institute of Psychology and Pedagogy, Clinical and Biological Psychology, Ulm, 89081, Germany

^4^Ludwig-Maximilians-Universität München, Department of Neurology, Munich, 80539, Germany

^5^SM della Pietà General Hospital, Neurology Unit, Nola, 80035, Italy

^6^University of Campania “Luigi Vanvitelli”, Department of Psychology, Caserta, 81100, Italy

*[luigi.TROJANO@unicampania.it](mailto:luigi.TROJANO@unicampania.it)

^+^These authors contributed equally to this work.

**Supplementary Figure S1.** *Flow-chart of patient selection for the study.* Abbreviations: DoC = Disorders of Consciousness


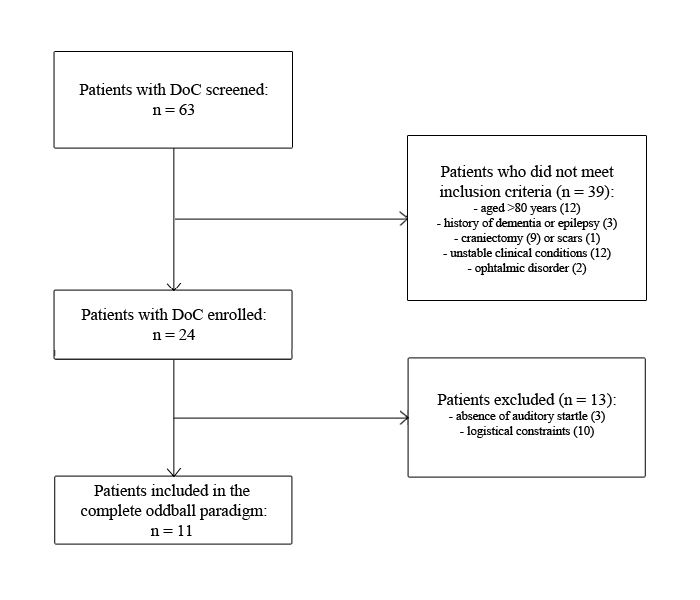


**Supplementary Table S1.** Spearman’s ρ (*p*-values) for patients’ eye blink rate (EBR) collected across the different phases and tasks. *p*-values are adjusted with the false discovery rate correction. Significant *p*-values are reported in bold.

|  |  | Passive | | | Active | | | |
| --- | --- | --- | --- | --- | --- | --- | --- | --- |
|  |  | Rest 1 | Oddball | Rest 2 | Rest 1 | Oddball | | Rest 2 |
| Passive | Rest 1 | - | .90 (<**.001**) | .85 (**.005**) | .69 **(**.06) | .83 (**.009**) | .74 (**.04**) | |
|  | Oddball | - | - | .88 (<**.001**) | .57 (.15) | .69 (.06) | .65 (.08) | |
|  | Rest 2 | - | - | - | .51 (.20) | .65 (**.**08) | .57 (.15) | |
| Active | Rest 1 | - | - | - | - | .90 (**<.001**) | .98 (**<.001**) | |
|  | Oddball | - | - | - | - | - | .91 (**<.001**) | |
|  | Rest 2 | - | - | - | - | - | - | |

**Supplementary Table S2.** Average across midline channels (Fz, Cz, Pz) of the P300 peak amplitude and latency values for each patient.

|  |  | Passive | | | Active | | |
| --- | --- | --- | --- | --- | --- | --- | --- |
| Patient | Diagnosis | Accepted epochs | Amplitude (µV) | Latency (ms) | Accepted epochs | Amplitude (µV) | Latency (ms) |
| 1 | VS/UWS | 297 | 1.93 | 434.91 | 136 | 2.57 | 395.45 |
| 3 | VS/UWS | 367 | 0.60 | 275.93 | 338 | 0.32 | 304.77 |
| 5 | VS/UWS | 266 | -0.02 | 467.63 | 387 | 2.12 | 436.26 |
| 12 | MCS+ | 234 | 4.92 | 388.64 | 151 | 4.71 | 478.70 |
| 14 | MCS- | 70 | 4.17 | 311.28 | 51 | 0.57 | 306.06 |
| 15 | MCS- | 36 | 2.73 | 262.27 | 50 | 2.1 | 315.97 |
| 16 | MCS+ | 87 | 0.08 | 332.65 | 193 | 0.96 | 332.26 |
| 17 | MCS- | 184 | 2.06 | 410.38 | 109 | 1.65 | 457.22 |
| 18 | MCS- | 51 | 4.29 | 372.05 | 119 | 2.07 | 316.18 |
| 20 | MCS- | 281 | 2.6 | 349.72 | 152 | 2.8 | 307.87 |
